# Hepatic Epithelioid Hemangioendothelioma: A Rare Vascular Neoplasm of the Liver

**DOI:** 10.7759/cureus.88941

**Published:** 2025-07-28

**Authors:** Cherechi O Nwabueze, Efe Erhus, Abhin Sapkota, Su Yeon Yeon, Anas Almoghrabi

**Affiliations:** 1 Internal Medicine, John H. Stroger, Jr. Hospital of Cook County, Chicago, USA; 2 Public Health, George Washington University, Washington, D.C., USA; 3 Medicine, University of Nigeria, Nsukka, NGA; 4 Pathology, University of Illinois at Chicago, Chicago, USA; 5 Gastroenterology and Hepatology, John H. Stroger, Jr. Hospital of Cook County, Chicago, USA

**Keywords:** epithelioid hemangioendothelioma, gastroenterology, gastrointestinal oncology, malignancy, rare liver disease

## Abstract

Epithelioid hemangioendothelioma (EHE) is a rare vascular neoplasm that can involve multiple organs. Most cases are asymptomatic and detected incidentally, but the clinical course can vary significantly. A 40-year-old man presented with abdominal and chest pain; imaging revealed large hepatic masses along with pulmonary nodules. Histopathological examination confirmed the diagnosis of EHE, with positivity for endothelial markers. Given the extent of the disease, systemic therapy was planned. This case highlights the challenges in diagnosing EHE and underscores the need for an individualized approach to managing this rare tumor.

## Introduction

Epithelioid hemangioendothelioma (EHE) is a rare, intermediate-grade vascular neoplasm, with an estimated global prevalence of fewer than one case per million people [1-3]. It most commonly affects individuals in their fourth to sixth decades of life, although cases have also been reported in both children and the elderly [4,5]. The liver, lungs, and bones are the most frequently involved sites, although EHE can also affect other areas, including the skin, meninges, and brain [1]. The clinical course of EHE is highly variable, ranging from indolent to aggressive [3,5]. The mean survival is approximately four years, with significantly higher mortality observed in cases involving both the liver and lungs [1]. Many cases are discovered incidentally during routine health screenings. In this report, we describe a case of hepatic and pulmonary EHE identified in an otherwise healthy young man.

## Case presentation

A 40-year-old gentleman with a past medical history of chronic low back pain presented to the emergency department with worsening abdominal and chest pain. He reported intermittent episodes of right upper quadrant (RUQ) and epigastric abdominal pain over the preceding nine months. Each episode lasted two to three days before resolving, and the pain worsened with the intake of fatty foods, without any identifiable relieving factors. He also endorsed right-sided chest pain that was in close proximity to the abdominal discomfort. Additionally, he reported occasional episodes of shortness of breath triggered by agitation or exertion. Over the past two weeks, he experienced an unintentional weight loss of 20 pounds.

He denied nausea, vomiting, diarrhea, constipation, hematemesis, melena, or hematochezia. He had no history of prior surgeries, and he denied the use of herbal supplements or over-the-counter pain medications. He was a nonsmoker and reported occasional alcohol consumption, typically drinking a six-pack of beer and one bottle of tequila every two months.

At the time of admission, his vital signs were within normal limits, and physical examination findings were unremarkable. Laboratory investigations revealed abnormal liver function tests and mild microcytic anemia. Detailed laboratory parameters with corresponding reference ranges are shown in Table 1.

**Table 1:** Laboratory parameters of the patient with corresponding reference ranges

Laboratory parameter	Observed value	Reference range
Hemoglobin	12.0 (low)	12.9-16.8 g/dL
Mean corpuscular volume	76.3 (low)	81.9-97.8 fL
Total leukocyte count	6	4.4-10.6 × 10³/µL
Platelet count	299	161-369 × 10³/µL
Activated partial thromboplastin time	40.6 (high)	23.5-35.1 seconds
Prothrombin time	13.9	11.7-14.5 seconds
International normalized ratio	1.12	0.8-1.1
Serum urea	14	8-20 mg/dL
Serum creatinine	1	0.6-1.4 mg/dL
Serum sodium	143	135-145 mEq/L
Serum potassium	4.6	3.5-5.0 mEq/L
Alkaline phosphatase	300 (high)	20-120 U/L
Alanine aminotransferase	80 (high)	0-40 U/L
Aspartate aminotransferase	50 (high)	0-40 U/L
Albumin	3.8	3.8-5.2 g/dL
Total bilirubin	0.5	0.2-1.2 mg/dL
Lipase	18	5-55 U/L

**Figure 1:**
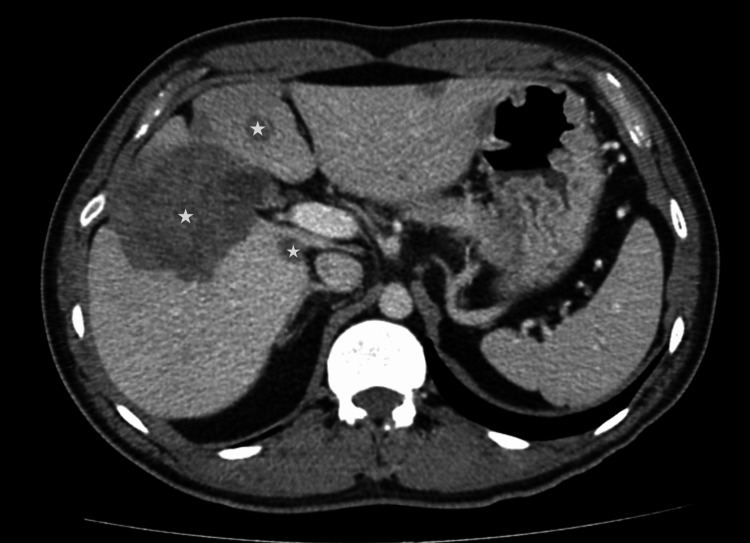
CT scan of the abdomen showing multiple hypoattenuating lesions throughout the liver ☆ indicates the hypoattenuating lesions.

A contrast-enhanced abdominopelvic CT scan was performed to evaluate the cause of abdominal pain and revealed multiple hypoattenuating lesions in the liver. The largest lesion, located peripherally in the right hepatic lobe, measured 7.8 × 3.3 cm, while another representative mass in the same lobe measured 6.5 × 6.5 cm (Figure 1).

A contrast-enhanced CT scan of the chest revealed numerous bilateral subcentimeter pulmonary nodules, raising suspicion for metastatic disease of unknown primary origin. Gastroenterology was consulted, and the patient initially underwent a colonoscopy, which revealed scattered diverticulosis but was otherwise unremarkable. This was followed by upper endoscopy, which showed only mild erythema in the gastric antrum and body.

**Figure 2:**
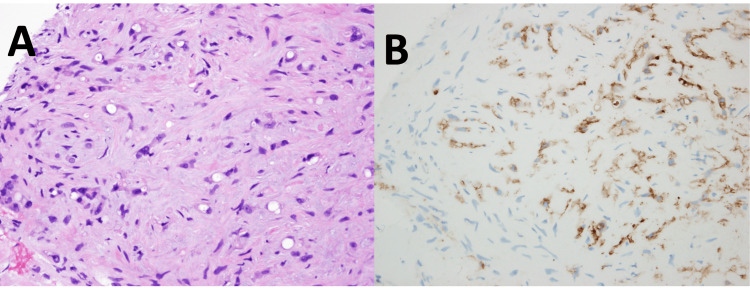
Histopathological slides of the liver biopsy positive for EHE (A) Tumor cells display epithelioid endothelial morphology with cytoplasmic vacuolation, some containing red blood cells within a fibromyxoid matrix (H&E, original magnification ×400). (B) Immunohistochemical staining for CD31 highlights primitive vascular structures (CD31, original magnification ×400). EHE, epithelioid hemangioendothelioma

An ultrasound-guided liver biopsy was subsequently performed by interventional radiology. The biopsy sample from the right hepatic lobe lesion demonstrated epithelioid cells with cytoplasmic vacuoles, some containing red blood cells, within a fibromyxoid stroma. Immunohistochemical staining showed tumor cells positive for ERG, CD31, and factor VIII, confirming the vascular origin of the tumor. There was also focal, weak positivity for AE1/AE3, consistent with a diagnosis of EHE. Figure 2 shows the histopathologic and immunohistochemical findings of the liver biopsy positive for EHE.

Due to the extent of the disease with multifocal involvement and capsular invasion, palliative radiation was deemed to offer only transient pain relief and carried a significant risk of radiation-induced liver disease. Liver transplantation could have been a viable treatment option; however, the patient was not eligible due to his citizenship status. Furthermore, the presence of pulmonary metastases further disqualified him from transplant consideration. As a result, he was referred for outpatient follow-up with medical oncology to discuss the initiation of systemic therapy.

At his follow-up visit in the medical oncology clinic, he reported persistent but intermittent RUQ pain, which was controlled with acetaminophen. A treatment plan was made to initiate therapy with bevacizumab and capecitabine.

## Discussion

EHE is often diagnosed incidentally in patients who present to the hospital for unrelated reasons. In symptomatic individuals, clinical manifestations usually correlate with the location of the tumor. Our patient reported RUQ pain, chest pain, and weight loss and was subsequently found to have large hepatic masses and multiple pulmonary lesions. Hepatic EHE typically presents with RUQ discomfort and weight loss, as seen in this case [6]. In contrast, pulmonary and pleural EHE may manifest with cough, dyspnea, or pleural effusion, none of which were observed in our patient [7].

Although imaging may reveal distinct lesions in commonly affected organs, certain CT and MRI features - such as capsular retraction, the “lollipop sign,” and the “target sign” - are considered relatively specific for hepatic EHE [8-10]. However, these imaging features were not identified in this case. A definitive diagnosis of EHE requires a combination of histopathological evaluation, immunohistochemical staining, and molecular genetic testing. The classic histological hallmark is the presence of epithelioid endothelial cells embedded in a myxoid or fibrotic stroma, which was evident in our patient [11]. Additionally, tumor cells often exhibit immunoreactivity for endothelial markers such as ERG, CD31, CD34, and factor VIII-related antigen [11-14]. In this case, immunohistochemical staining was positive for ERG and CD31. Expression of CAMTA1 is highly specific for EHE and helps differentiate it from other epithelioid vascular tumors. The most common genetic alteration in EHE is the *WWTR1-CAMTA1* fusion gene, identified in approximately 90% of cases. A less common fusion, *YAP1-TFE3*, occurs in fewer than 5% of cases and is typically associated with more vasoformative histologic features [13].

Management of EHE depends on the extent and location of the disease. The American College of Gastroenterology recommends surgical resection for localized hepatic EHE, with reported cure rates of 70-80% [7]. Liver transplantation may be an option for patients with extensive hepatic involvement. For unresectable or inoperable liver-confined disease, locoregional treatments such as ablation or external beam radiotherapy can be considered [14]. Systemic therapies are generally reserved for patients with progressive, unresectable disease. In selected cases, observation with active surveillance may be appropriate, as some patients demonstrate stable disease without progression. However, no standardized criteria currently exist to identify individuals suitable for surveillance alone [15].

## Conclusions

EHE is a rare and diagnostically challenging vascular tumor, often discovered incidentally during the evaluation of unrelated symptoms. When present, clinical manifestations typically correspond to the anatomical distribution of the disease. This case underscores the importance of including EHE in the differential diagnosis of hepatic and pulmonary masses, particularly when symptoms are nonspecific. Although imaging may raise suspicion for EHE, a definitive diagnosis relies on histopathological and immunohistochemical evaluation of liver biopsy samples. Management should be tailored to the extent of the disease, with treatment options ranging from close observation and surgical resection to liver transplantation, locoregional therapies, and systemic treatment.
